# Response to letter for critiques on our published article: Effect of tracheal tube cuff inflation with alkalinized lidocaine versus air on hemodynamic responses during extubation and post-operative airway morbidities in children: prospective observational cohort study, Ethiopia

**DOI:** 10.1186/s12871-023-02085-1

**Published:** 2023-04-24

**Authors:** Biniam Assefa, Hirbo Samuel, Fissiha Fentie, Tenbite Daniel, Assefa Hika, Bacha Aberra, Belete Alemu

**Affiliations:** 1 Department of Anesthesia, College of Medicine and Health Sciences, Addis Ababa University, Addis Ababa, Ethiopia; 2 Department of Anesthesia, College of Health Science, Aksum University, Aksum, Ethiopia; 3 Department of Anesthesia, College of Health Science, Hawassa University, Hawassa, Ethiopia; 4 University Hospital, Hiwot Fana Specialized University Hospital, Harar, Ethiopia

**Keywords:** Endotracheal tube, Pre-operative Airway Assessment, Postoperative sore throat

## Abstract

Endotracheal tube with an inflated cuff was used to manage and maintain the airway during general anesthesia in children. When the lateral pressure exerted by an inflated Endotracheal tube cuff on tracheal mucosa exceeds capillary perfusion pressure, patients may complain of cough, sore throat, and hoarseness in the postoperative period.

First of all, we would like to thank you for your email.

This research is done in a resource-limited setting, where getting advanced anesthesia equipment are challenging; but we anesthetist and researchers are always striving to do our best with what we have at hand and doing research to contribute to the scientific community.

As it is the first research in the study setting, this research help as a baseline for future researcher.

We answer the raised questions as follows:

1. The answer to the first question.

In our study setting pre-anesthesia evaluation and airway assessment were done a day before surgery for all pediatric patients who underwent elective surgery and, all patients were also visited in the morning before surgery. In this study, an airway assessment was performed and no difficult airway was noticed. Moreover, patients with a difficult airway, especially pediatric patients with a difficult airway, are usually not included in prospective studies.

In the methods part, under the procedure section, we already describe direct Laryngoscopy was performed and the trachea was intubated with a standard cuffed endotracheal tube, which is a Murphy, white PVC ETT. A video laryngoscope is not available in our environment.

In this study, stylets were used for each intubation, and there was one intubation attempt for all study participants.

2. The answer to the second question.

In this study, extubation followed common criteria for awake extubation in children: facial grimace, eye-opening, end-tidal isoflurane concentration less than 0.15%, spontaneous tidal volume greater than 5ml/kg, conjugate gaze, purposeful movement, movement other than coughing, positive laryngeal stimulation test, and oxygen saturation greater than 97% [1].

In this study, suctioning was performed before and during extubation for all study participants. It was difficult to measure the pressure of suction because our suction devices are not advanced and are not equipped with a precise pressure gauge to detect small pressure differences.

In the Methods chapter, under the operational definition section, we already operationalize sore throat as a constant pain or discomfort in the throat independent of swallowing. The degree of postoperative sore throat or other postoperative airway morbidities was not described because they were neither the primary nor the secondary objective of our study. This study focused on hemodynamic responses during extubation and the incidence of postoperative airway morbidities. The Authors can answer your question about the severity of sore throat that study participants graded the severity of a sore throat using the FLACC score [2] for ages 3–7 and VAS score [3] for ages 8–13. In all case, if there is any pain or discomfort, our data collector report to the attending nurses to take further action accordingly.

3. The answer to the third question.

Opioids used during anesthesia and extubation may indeed reduce hemodynamic fluctuation and cough response. In this study, an identical analgesia protocol was used for both groups of patients, so the influence of the type and amount of analgesics on the results of the study was avoided.

Sufentanil and remifentanil are not available in our study setting. we use Fentanyl as an induction opioid and analgesia was maintained with IV paracetamol. All study participant takes IV fentanyl 2 µg/kg during induction and 15 mg/kg IV paracetamol for maintenance, since the protocol was identical in the studied groups, we did not report this.

4. The answer to the fourth question.

Patient satisfaction and postoperative QoR-15 are undoubtedly important parts of anesthesiology practice. However, this was neither the primary nor the secondary goal of this study, which is why it was not presented. During the 24 h of data collection, your data assessors had a conversation with the study participants about their satisfaction and comfort after surgery. Almost all study participants and their parents stated they were satisfied and comfortable.

## Data Availability

Not applicable.
